# The species *Severe acute respiratory syndrome-related coronavirus*: classifying 2019-nCoV and naming it SARS-CoV-2

**DOI:** 10.1038/s41564-020-0695-z

**Published:** 2020-03-02

**Authors:** Coronaviridae Study Group of the International Committee on Taxonomy of Viruses, Alexander E. Gorbalenya, Susan C. Baker, Ralph S. Baric, Raoul J. de Groot, Christian Drosten, Anastasia A. Gulyaeva, Bart L. Haagmans, Chris Lauber, Andrey M. Leontovich, Benjamin W. Neuman, Dmitry Penzar, Stanley Perlman, Leo L. M. Poon, Dmitry V. Samborskiy, Igor A. Sidorov, Isabel Sola, John Ziebuhr

**Affiliations:** 1 Department of Biomedical Data Sciences, Leiden University Medical Center, Leiden, the Netherlands; 2 Department of Medical Microbiology, Leiden University Medical Center, Leiden, the Netherlands; 3 Faculty of Bioengineering and Bioinformatics and Belozersky Institute of Physico-Chemical Biology, Lomonosov Moscow State University, Moscow, Russia; 4 Department of Microbiology and Immunology, Loyola University of Chicago, Stritch School of Medicine, Maywood, IL USA; 5 Department of Epidemiology, University of North Carolina, Chapel Hill, NC USA; 6 Division of Virology, Department of Biomolecular Health Sciences, Faculty of Veterinary Medicine, Utrecht University, Utrecht, the Netherlands; 7 Institute of Virology, Charité – Universitätsmedizin Berlin, Berlin, Germany; 8 Viroscience Lab, Erasmus MC, Rotterdam, the Netherlands; 9 Texas A&M University-Texarkana, Texarkana, TX USA; 10 Department of Microbiology and Immunology, University of Iowa, Iowa City, IA USA; 11 Centre of Influenza Research & School of Public Health, The University of Hong Kong, Hong Kong, People’s Republic of China; 12 Department of Molecular and Cell Biology, National Center of Biotechnology (CNB-CSIC), Campus de Cantoblanco, Madrid, Spain; 13 Institute of Medical Virology, Justus Liebig University Giessen, Giessen, Germany

**Keywords:** Microbiology, Biodiversity, Diseases, Virology, Applied microbiology

## Abstract

The present outbreak of a coronavirus-associated acute respiratory disease called coronavirus disease 19 (COVID-19) is the third documented spillover of an animal coronavirus to humans in only two decades that has resulted in a major epidemic. The *Coronaviridae* Study Group (CSG) of the International Committee on Taxonomy of Viruses, which is responsible for developing the classification of viruses and taxon nomenclature of the family *Coronaviridae*, has assessed the placement of the human pathogen, tentatively named 2019-nCoV, within the *Coronaviridae*. Based on phylogeny, taxonomy and established practice, the CSG recognizes this virus as forming a sister clade to the prototype human and bat severe acute respiratory syndrome coronaviruses (SARS-CoVs) of the species *Severe acute respiratory syndrome-related coronavirus*, and designates it as SARS-CoV-2. In order to facilitate communication, the CSG proposes to use the following naming convention for individual isolates: SARS-CoV-2/host/location/isolate/date. While the full spectrum of clinical manifestations associated with SARS-CoV-2 infections in humans remains to be determined, the independent zoonotic transmission of SARS-CoV and SARS-CoV-2 highlights the need for studying viruses at the species level to complement research focused on individual pathogenic viruses of immediate significance. This will improve our understanding of virus–host interactions in an ever-changing environment and enhance our preparedness for future outbreaks.

Upon a viral outbreak, it is important to rapidly establish whether the outbreak is caused by a new or a previously known virus (Box 1), as this helps decide which approaches and actions are most appropriate to detect the causative agent, control its transmission and limit potential consequences of the epidemic. The assessment of virus novelty also has implications for virus naming and, on a different timescale, helps to define research priorities in virology and public health.

## Box 1 Virus discovery and naming: from disease-based to phenotype-free

Understanding the cause of a specific disease that spreads among individuals of the same host species (infectivity) was the major driving force for the discovery of the first virus in plants, and subsequently many others in all forms of life, including humans. Historically, the range of diseases and hosts that specific viruses are associated with have been the two key characteristics used to define viruses, given that they are invisible to the naked eye^46^. Viral phenotypic features include those that, like a disease, are predominantly shaped by virus–host interactions including transmission rate or immune correlates of protection, and others that are largely virus-specific, such as the architecture of virus particles. These features are of critical importance to control, and respond to medically and economically important viruses — especially during outbreaks of severe disease — and dominate the general perception of viruses.

However, the host of a given virus may be uncertain, and virus pathogenicity remains unknown for a major (and fast-growing) proportion of viruses, including many coronaviruses discovered in metagenomics studies using next-generation sequencing technology of environmental samples^47,48^. These studies have identified huge numbers of viruses that circulate in nature and have never been characterized at the phenotypic level. Thus, the genome sequence is the only characteristic that is known for the vast majority of viruses, and needs to be used in defining specific viruses. In this framework, a virus is defined by a genome sequence that is capable of autonomous replication inside cells and dissemination between cells or organisms under appropriate conditions. It may or may not be harmful to its natural host. Experimental studies may be performed for a fraction of known viruses, while computational comparative genomics is used to classify (and deduce characteristics of) all viruses. Accordingly, virus naming is not necessarily connected to disease but rather informed by other characteristics.

In view of the above advancements and when confronted with the question of whether the virus name for the newly identified human virus should be linked to the (incompletely defined) disease that this virus causes, or rather be established independently from the virus phenotype, the CSG decided to follow a phylogeny-based line of reasoning to name this virus whose ontogeny can be traced in the figure in Box 1.

**Figure:**
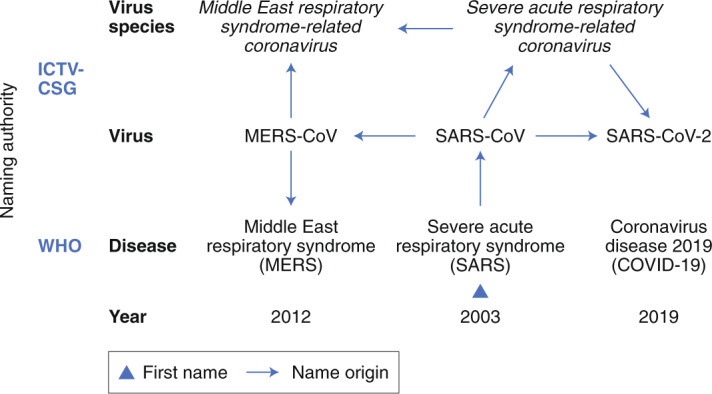
History of coronavirus naming during the three zoonotic outbreaks in relation to virus taxonomy and diseases caused by these viruses. According to the current international classification of diseases^49^, MERS and SARS are classified as 1D64 and 1D65, respectively.

For many human virus infections such as influenza virus^1^ or norovirus^2^ infections, well-established and internationally approved methods, standards and procedures are in place to identify and name the causative agents of these infections and report this information promptly to public health authorities and the general public. In outbreaks involving newly emerged viruses, the situation may be different, and appropriate procedures to deal with these viruses need to be established or refined with high priority.

Here, we present an assessment of the genetic relatedness of the newly identified human coronavirus^3^, provisionally named 2019-nCoV, to known coronaviruses, and detail the basis for (re)naming this virus severe acute respiratory syndrome coronavirus 2 (SARS-CoV-2), which will be used hereafter. Given the public interest in naming newly emerging viruses and the diseases caused by these viruses in humans, we will give a brief introduction to virus discovery and classification — specifically the virus species concept — and the roles of different bodies, such as the World Health Organization (WHO) and the International Committee on Taxonomy of Viruses (ICTV), in this process. We hope this will help readers to better understand the scientific approach we have taken to arrive at this name, and we will also discuss implications of this analysis and naming decision.

## Classifying and naming viruses and virus species

Defining the novelty of viruses is one of the topics that virus classification deals with. The classification of RNA viruses needs to consider their inherent genetic variability, which often results in two or more viruses with non-identical but similar genome sequences being regarded as variants of the same virus. This immediately poses the question of how much difference to an existing group is large enough to recognize the candidate virus as a member of a new, distinct group. This question is answered in best practice by evaluating the degree of relatedness of the candidate virus to previously identified viruses infecting the same host or established monophyletic groups of viruses, often known as genotypes or clades, which may or may not include viruses of different hosts. This is formally addressed in the framework of the official classification of virus taxonomy and is overseen and coordinated by the ICTV^4^. Viruses are clustered in taxa in a hierarchical scheme of ranks in which the species represents the lowest and most populous rank containing the least diverged groups (taxa) of viruses (Box 2). The ICTV maintains a Study Group for each virus family. The Study Groups are responsible for assigning viruses to virus species and taxa of higher ranks, such as subgenera, genera and subfamilies. In this context they play an important role in advancing the virus species concept and highlighting its significance^5^.

Virus nomenclature is a formal system of names used to label viruses and taxa. The fact that there are names for nearly all viruses within a species is due to the historical perception of viruses as causative agents of specific diseases in specific hosts, and to the way we usually catalogue and classify newly discovered viruses, which increasingly includes viruses that have not been linked to any known disease in their respective hosts (Box 1). The WHO, an agency of the United Nations, coordinates international public health activities aimed at combating, containing and mitigating the consequences of communicable diseases—including major virus epidemics—and is responsible for naming disease(s) caused by newly emerging human viruses. In doing so, the WHO often takes the traditional approach of linking names of specific diseases to viruses (Box 1) and assessing virus novelty by an apparent failure to detect the causative agent using established diagnostic assays.

Apart from disease, geography and the organism from which a given virus was isolated also dominate the nomenclature, occasionally engraving connections that may be accidental (rather than typical) or even stigmatizing, which should be avoided. Establishing a universal nomenclature for viruses was one of the major tasks of the ICTV when it was founded more than 50 years ago^4^. When the species rank was established in the taxonomy of viruses^6^, ICTV’s responsibility for naming viruses was shifted to naming and establishing species. ICTV Study Groups may also be involved in virus naming on a case-by-case basis as an extension of their official remit, as well as using the special expertise of their members. As virus species names are often very similar to the name of the founding member of the respective species, they are frequently confused in the literature with names of individual viruses in this species. The species name is italicized, starts with a capital letter and should not be spelled in an abbreviated form^7^; hence the species name *Severe acute respiratory syndrome-related coronavirus*. In contrast, this convention does not apply to virus names, hence severe acute respiratory syndrome coronavirus, or SARS-CoV, as it is widely known.

Box 2 Identifying viral species The terms strain and isolate are commonly used to refer to virus variants, although there are different opinions as to which term should be used in a specific context. If a candidate virus clusters within a known group of isolates, it is a variant of this group and may be considered as belonging to this known virus group. In contrast, if the candidate virus is outside of known groups and its distances to viruses in these groups are comparable to those observed between viruses of different groups (intergroup distances), the candidate virus is distinct and can be considered novel.

This evaluation is usually conducted in silico using phylogenetic analysis, which may be complicated by uneven rates of evolution that vary across different virus lineages and genomic sites due to mutation, including the exchange of genome regions between closely related viruses (homologous recombination). However, given that the current sampling of viruses is small and highly biased toward viruses of significant medical and economic interest, group composition varies tremendously among different viruses, making decisions on virus novelty group-specific and dependent on the choice of the criteria selected for this assessment.

These challenges are addressed in the framework of virus taxonomy, which partitions genomic variation above strain or isolate level and develops a unique taxon nomenclature under the supervision of the ICTV^4,5^. To decide on whether a virus represents a new species—that is, the least diverged (and most populated) group of viruses—taxonomists use the results of different analyses. Taxonomical classification is hierarchical, using nested groups (taxa) that populate different levels (ranks) of classification. Taxa of different ranks differ in their intra-taxon pairwise divergence, which increases from the smallest at the species rank to the largest at the realm rank^30^. They may also be distinguished by taxon-specific markers that characterize natural groupings. Only the species and genus ranks need to be specified to classify a new virus; filling other ranks is optional. If a virus prototypes a new species, it will be regarded as taxonomically novel. If (within this framework) a virus crosses a host barrier and acquires novel properties, its classification will not change (that is, it remains part of the original species) even if the virus establishes a permanent circulation in the new host, which likely happened with coronaviruses of the four species that circulate in humans and display seasonal peaks (reviewed in ref. ^50^). Importantly, the criteria used to define a viral species in one virus family such as *Coronaviridae* may not be applicable to another family such as *Retroviridae*, and vice versa, since Study Groups are independent in their approach to virus classification.

## Defining the place of SARS-CoV-2 within the *Coronaviridae*

Researchers studying coronaviruses—a family of enveloped positive-strand RNA viruses infecting vertebrates^8^—have been confronted several times with the need to define whether a newly emerged virus causing a severe or even life-threatening disease in humans belongs to an existing or a new (yet-to-be-established) species. This happened with SARS^9–12^ and with Middle East respiratory syndrome (MERS)^13,14^ a few years later. Each time, the virus was placed in the taxonomy using information derived from a sequence-based family classification^15,16^.

**Fig. 1:**
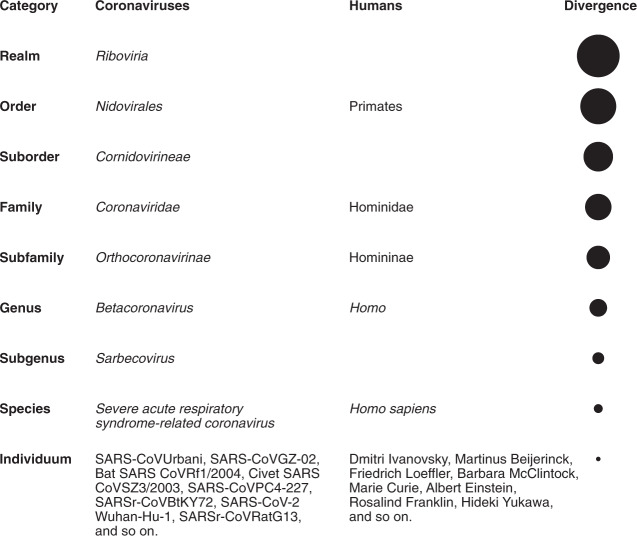
Taxonomy of selected coronaviruses. Shown is the full taxonomy of selected coronaviruses in comparison with the taxonomy of humans (the founders of virology and other eminent scientists represent individual human beings for the sake of this comparison), which is given only for categories (ranks) that are shared with the virus taxonomy. Note that these two taxonomies were independently developed using completely different criteria. Although no equivalence is implied, the species of coronaviruses is interpreted sensu stricto as accepted for the species of humans.

The current classification of coronaviruses recognizes 39 species in 27 subgenera, five genera and two subfamilies that belong to the family *Coronaviridae*, suborder *Cornidovirineae*, order *Nidovirales* and realm *Riboviria*^17–19^ (Fig. 1). The family classification and taxonomy are developed by the *Coronaviridae* Study Group (CSG), a working group of the ICTV^20^. The CSG is responsible for assessing the place of new viruses through their relation to known viruses in established taxa, including placements relating to the species *Severe acute respiratorysyndrome-related coronavirus*. In the classification of nidoviruses, species are considered biological entities demarcated by a genetics-based method^21^, while generally virus species are perceived as man-made constructs^22^. To appreciate the difference between a nidoviral species and the viruses grouped therein, it may be instructive to look at their relationship in the context of the full taxonomy structure of several coronaviruses. Although these viruses were isolated at different times and locations from different human and animal hosts (with and without causing clinical disease), they all belong to the species *Severe acute respiratorysyndrome-related coronavirus*, and their relationship parallels that between human individuals and the species *Homo sapiens* (Fig. 1).

**Fig. 2:**
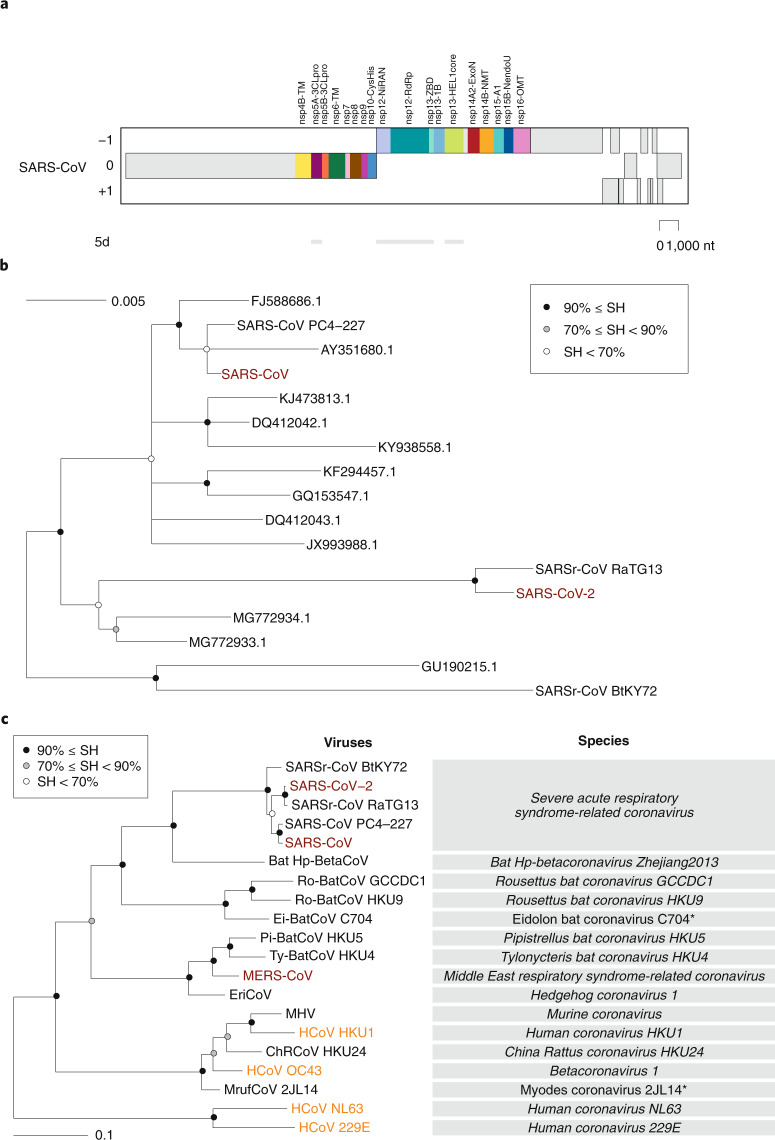
Phylogeny of coronaviruses. **a**, Concatenated multiple sequence alignments (MSAs) of the protein domain combination^44^ used for phylogenetic and DEmARC analyses of the family *Coronaviridae*. Shown are the locations of the replicative domains conserved in the ordert *Nidovirales* in relation to several other ORF1a/b-encoded domains and other major ORFs in the SARS-CoV genome. 5d, 5 domains: nsp5A-3CLpro, two beta-barrel domains of the 3C-like protease; nsp12-NiRAN, nidovirus RdRp-associated nucleotidyltransferase; nsp12-RdRp, RNA-dependent RNA polymerase; nsp13-HEL1 core, superfamily 1 helicase with upstream Zn-binding domain (nsp13-ZBD); nt, nucleotide. **b**, The maximum-likelihood tree of SARS-CoV was reconstructed by IQ‑TREE v.1.6.1 (ref. ^45^) using 83 sequences with the best fitting evolutionary model. Subsequently, the tree was purged from the most similar sequences and midpoint-rooted. Branch support was estimated using the Shimodaira–Hasegawa (SH)-like approximate likelihood ratio test with 1,000 replicates. GenBank IDs for all viruses except four are shown; SARS-CoV, AY274119.3; SARS-CoV-2, MN908947.3; SARSr-CoV_BtKY72, KY352407.1; SARS-CoV_PC4-227, AY613950.1. **c**, Shown is an IQ‑TREE maximum-likelihood tree of single virus representatives of thirteen species and five representatives of the species *Severe acute respiratory syndrome-related coronavirus* of the genus *Betacoronavirus*. The tree is rooted with HCoV-NL63 and HCoV-229E, representing two species of the genus *Alphacoronavirus*. Purple text highlights zoonotic viruses with varying pathogenicity in humans; orange text highlights common respiratory viruses that circulate in humans. Asterisks indicate two coronavirus species whose demarcations and names are pending approval from the ICTV and, thus, these names are not italicized.

Even without knowing anything about the species concept, every human recognizes another human as a member of the same species. However, for assigning individual living organisms to most other species, specialized knowledge and tools for assessing inter-individual differences are required. The CSG uses a computational framework of comparative genomics^23^, which is shared by several ICTV Study Groups responsible for the classification and nomenclature of the order *Nidovirales* and coordinated by the ICTV *Nidovirales* Study Group (NSG)^24^ (Box 3). The Study Groups quantify and partition the variation in the most conserved replicative proteins encoded in open reading frames 1a and 1b (ORF1a/1b) of the coronavirus genome (Fig. 2a) to identify thresholds on pair-wise patristic distances (PPDs) that demarcate virus clusters at different ranks.

Consistent with previous reports, SARS-CoV-2 clusters with SARS-CoVs in trees of the species *Severe acute respiratory syndrome-related coronavirus* (Fig. 2b) and the genus *Betacoronavirus* (Fig. 2c)^25–27^. Distance estimates between SARS-CoV-2 and the most closely related coronaviruses vary among different studies depending on the choice of measure (nucleotide or amino acid) and genome region. Accordingly, there is no agreement yet on the exact taxonomic position of SARS-CoV-2 within the subgenus *Sarbecovirus*. When we included SARS-CoV-2 in the dataset used for the most recent update (May 2019) of the coronavirus taxonomy currently being considered by ICTV^19^, which includes 2,505 coronaviruses, the species composition was not affected and the virus was assigned to the species *Severe acute respiratory syndrome-related coronavirus*, as detailed in Box 4.

With respect to novelty, SARS-CoV-2 differs from the two other zoonotic coronaviruses, SARS-CoV and MERS-CoV, introduced to humans earlier in the twenty-first century. Previously, the CSG established that each of these two viruses prototype a new species in a new informal subgroup of the genus *Betacoronavirus*^15,16^. These two informal subgroups were recently recognized as subgenera *Sarbecovirus* and *Merbecovirus*^18,28,29^ when the subgenus rank was established in the virus taxonomy^30^. Being the first identified representatives of a new species, unique names were introduced for the two viruses and their taxa in line with the common practice and state of virus taxonomy at the respective times of isolation. The situation with SARS-CoV-2 is fundamentally different because this virus is assigned to an existing species that contains hundreds of known viruses predominantly isolated from humans and diverse bats. All these viruses have names derived from SARS-CoV, although only the human isolates collected during the 2002–2003 outbreak have been confirmed to cause SARS in infected individuals. Thus, the reference to SARS in all these virus names (combined with the use of specific prefixes, suffixes and/or genome sequence IDs in public databases) acknowledges the phylogenetic (rather than clinical disease-based) grouping of the respective virus with the prototypic virus in that species (SARS-CoV). The CSG chose the name SARS-CoV-2 based on the established practice for naming viruses in this species and the relatively distant relationship of this virus to the prototype SARS-CoV in a species tree and the distance space (Fig. 2b and the figure in Box 4).

The available yet limited epidemiological and clinical data for SARS-CoV-2 suggest that the disease spectrum and transmission efficiency of this virus^31–35^ differ from those reported for SARS-CoV^9^. To accommodate the wide spectrum of clinical presentations and outcomes of infections caused by SARS-CoV-2 (ranging from asymptomatic to severe or even fatal in some cases)^31^, the WHO recently introduced a rather unspecific name (coronavirus disease 19, also known as COVID-19 (ref. ^36^)) to denote this disease. Also, the diagnostic methods used to confirm SARS-CoV-2 infections are not identical to those of SARS-CoV. This is reflected by the specific recommendations for public health practitioners, healthcare workers and laboratory diagnostic staff for SARS-CoV-2 (for example, the WHO guidelines for SARS-CoV-2 (ref. ^37^). By uncoupling the naming conventions used for coronaviruses and the diseases that some of them cause in humans and animals, we wish to support the WHO in its efforts to establish disease names in the most appropriate way (for further information, see the WHO’s guidelines for disease naming^38^). The further advancement of naming conventions is also important because the ongoing discovery of new human and animal viruses by next-generation sequencing technologies can be expected to produce an increasing number of viruses that do not (easily) fit the virus–disease model that was widely used in the pre-genomic era (Box 1). Having now established different names for the causative virus (SARS-CoV-2) and the disease (COVID-19), the CSG hopes that this will raise awareness in both the general public and public health authorities regarding the difference between these two entities. The CSG promotes this clear distinction because it will help improve the outbreak management and also reduces the risk of confusing virus and disease, as has been the case over many years with SARS-CoV (the virus) and SARS (the disease).

To facilitate good practice and scientific exchange, the CSG recommends that researchers describing new viruses (that is, isolates) in this species adopt a standardized format for public databases and publications that closely resembles the formats used for isolates of avian coronaviruses^39^, filoviruses^40^ and influenza virus^1^. The proposed naming convention includes a reference to the host organism that the virus was isolated from, the place of isolation (geographic location), an isolate or strain number, and the time of isolation (year or more detailed) in the format virus/host/location/isolate/date; for example, SARS-CoV-2/human/Wuhan/X1/2019. This complete designation along with additional and important characteristics, such as pathogenic potential in humans or other hosts, should be included in the submission of each isolate genome sequence to public databases such as GenBank. In publications, this name could be further extended with a sequence database ID—for example, SARS-CoV-2/human/Wuhan/X1/2019_XYZ12345 (fictional example)—when first mentioned in the text. We believe that this format will provide critical metadata on the major characteristics of each particular virus isolate (genome sequence) required for subsequent epidemiological and other studies, as well as for control measures.

Box 3 Classifying coronaviruses Initially, the classification of coronaviruses was largely based on serological (cross-) reactivities to the viral spike protein, but is now based on comparative sequence analyses of replicative proteins. The choice of proteins and the methods used to analyse them have gradually evolved since the start of this century^20,28,29,51^. The CSG currently analyses 3CLpro, NiRAN, RdRp, ZBD and HEL1 (ref. ^52^) (Fig. 2a), two domains less than previously used in the analyses conducted between 2009 and 2015 (refs. ^16,18^). According to our current knowledge, these five essential domains are the only ones conserved in all viruses of the order *Nidovirales*^52^. They are thus used for the classification by all ICTV nidovirus study groups (coordinated by the NSG).

Since 2011, the classification of coronaviruses and other nidoviruses has been assisted by the DivErsity pArtitioning by hieRarchical Clustering (DEmARC) software, which defines taxa and ranks^23,24^. Importantly, the involvement of all coronavirus genome sequences available at the time of analysis allows family-wide designations of demarcation criteria for all ranks, including species, regardless of the taxa sampling size, be it a single or hundreds of virus(es). DEmARC delineates monophyletic clusters (taxa) of viruses using weighted linkage clustering in the PPD space and according to the classification of ranks defined through clustering cost (CC) minima presented as PPD thresholds (PPD accounts for multiple substitutions at all sequence positions and thus may exceed 1.0, which is the limit for conventional pair-wise distances (PDs)). In the DEmARC framework, the persistence of thresholds in the face of increasing virus sampling is interpreted to reflect biological forces and environmental factors^21^. Homologous recombination, which is common in coronaviruses^53–55^, is believed to be restricted in genome regions encoding the most essential proteins, such as those used for classification, and to members of the same virus species. This restriction promotes intra-species diversity and contributes to inter-species separation. To facilitate the use of rank thresholds outside of the DEmARC framework, they are converted into PD and expressed as a percentage, which researchers commonly use to arrive at a tentative assignment of a given virus within the coronavirus taxonomy following conventional phylogenetic analysis of selected viruses.

Box 4 Classifying SARS-CoV-2 The species demarcation threshold (also known as demarcation limit) in the family *Coronaviridae* is defined by viruses whose PPD(s) may cross the inter-species demarcation PPD threshold (threshold ‘violators’). Due to their minute share of ~10^–4^ of the total number of all intra- and inter-species PPDs, these violators may not even be visually recognized in a conventional diagonal plot clustering viruses on a species basis (panel **a** of the figure in Box 4). Furthermore, they do not involve any virus of the species *Severe acute respiratory syndrome-related coronavirus*, as is evident from the analysis of maximal intraspecies PPDs of 2,505 viruses of all 49 coronavirus species (of which 39 are established and 10 are pending or tentative) (panel **b** of the figure in Box 4) and PDs of 256 viruses of this species (panel **c** of the figure in Box 4). Thus, the genomic variation of the known viruses of the species *Severe acute respiratory syndrome-related coronavirus* is smaller compared to that of other comparably well-sampled species—for example, those prototyped by MERS-CoV, human coronavirus OC43 (HCoV-OC43) and infectious bronchitis virus (IBV) (panel **b** of the figure in Box 4)—and this species is well separated from other known coronavirus species in the sequence space. Both of these characteristics facilitate the unambiguous assignment of SARS-CoV-2 to this species.

Intra-species PDs of SARS-CoV-2 belong to the top 25% of this species and also include the largest PD between SARS-CoV-2 and an African bat virus isolate (SARSr-CoV_BtKY72)^56^ (panel **c** of the figure in Box 4), representing two basal lineages within the species *Severe acute respiratory syndrome-related coronavirus* that constitute very few known viruses (Fig. 2b,c). These relationships stand in contrast to the shallow branching of the most populous lineage of this species, which includes all the human SARS-CoV isolates collected during the 2002–2003 outbreak and the closely related bat viruses of Asian origin identified in the search for the potential zoonotic source of that epidemic^57^. This clade structure is susceptible to homologous recombination, which is common in this species^44,58,59^; to formalize clade definition, it must be revisited after the sampling of viruses representing the deep branches has improved sufficiently. The current sampling defines a very small median PD for human SARS-CoVs, which is approximately 15 times smaller than the median PD determined for SARS-CoV-2 (0.16% versus 2.6%; panel **c** of the figure in Box 4). This small median PD of human SARS-CoVs also dominates the species-wide PD distribution (0.25%; panel **c** of the figure in Box 4).

**Figure:**
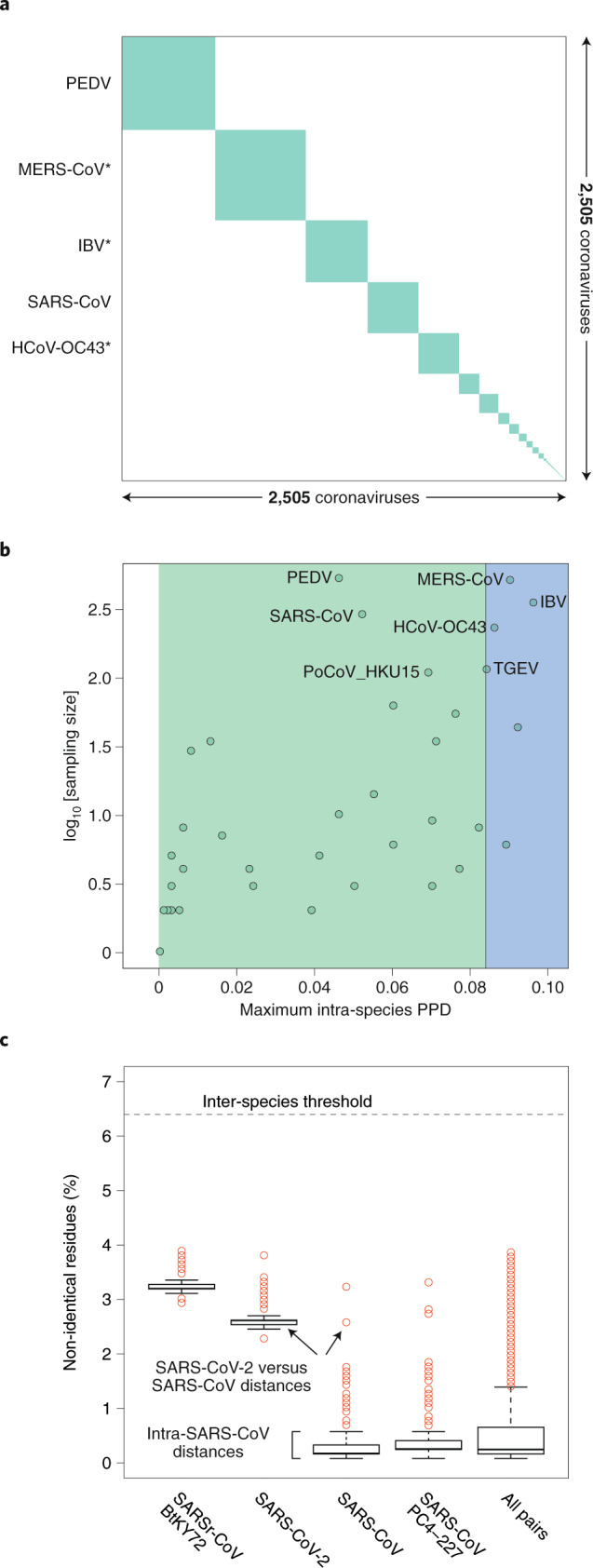
Pairwise distance demarcation of species in the family *Coronaviridae*. **a**, Diagonal matrix of PPDs of 2,505 viruses clustered according to 49 coronavirus species, 39 established and 10 pending or tentative, and ordered from the most to least populous species, from left to right; green and white, PPDs smaller and larger than the inter-species threshold, respectively. Areas of the green squares along the diagonal are proportional to the virus sampling of the respective species, and virus prototypes of the five most sampled species are specified to the left; asterisks indicate species that include viruses whose intra-species PPDs crossed the inter-species threshold (threshold ‘violators’). **b**, Maximal intra-species PPDs (*x* axis, linear scale) plotted against virus sampling (*y* axis, log scale) for 49 species (green dots) of the *Coronaviridae*. Indicated are the acronyms of virus prototypes of the seven most sampled species. Green and blue plot sections represent intra-species and intra-subgenera PPD ranges. The vertical black line indicates the inter-species threshold. **c**, Shown are the PDs of non-identical residues (*y* axis) for four viruses representing three major phylogenetic lineages (clades) of the species *Severe acute respiratorysyndrome-related coronavirus* (panel **b**) and all pairs of the 256 viruses of this species (‘all pairs’). The PD values were derived from pairwise distances in the MSA that were calculated using an identity matrix. Panels **a** and **b** were adopted from the DEmARC v.1.4 output.

## Expanding the focus from pathogens to virus species

Historically, public health and fundamental research have been focused on the detection, containment, treatment and analysis of viruses that are pathogenic to humans following their discovery (a reactive approach). Exploring and defining their biological characteristics in the context of the entire natural diversity as a species has never been a priority. The emergence of SARS-CoV-2 as a human pathogen in December 2019 may thus be perceived as completely independent from the SARS-CoV outbreak in 2002–2003. Although SARS-CoV-2 is indeed not a descendent of SARS-CoV (Fig. 2b), and the introduction of each of these viruses into humans was likely facilitated by independent unknown external factors, the two viruses are genetically so close to each other (Fig. 2c, panel **c** of the figure in Box 4) that their evolutionary histories and characteristics are mutually informative.

The currently known viruses of the species *Severe acute respiratory syndrome-related coronavirus* may be as (poorly) representative for this particular species as the few individuals that we selected to represent *H. sapiens* in Fig. 1. It is thus reasonable to assume that this biased knowledge of the natural diversity of the species *Severe acute respiratory syndrome-related coronavirus* limits our current understanding of fundamental aspects of the biology of this species and, as a consequence, our abilities to control zoonotic spillovers to humans. Future studies aimed at understanding the ecology of these viruses and advancing the accuracy and resolution of evolutionary analyses^41^ would benefit greatly from adjusting our research and sampling strategies. This needs to include an expansion of our current research focus on human pathogens and their adaptation to specific hosts to other viruses in this species. To illustrate the great potential of species-wide studies, it may again be instructive to draw a parallel to *H. sapiens*, and specifically to the impressive advancements in personalized medicine in recent years. Results of extensive genetic analyses of large numbers of individuals representing diverse populations from all continents have been translated into clinical applications and greatly contribute to optimizing patient-specific diagnostics and therapy. They were instrumental in identifying reliable predictive markers for specific diseases as well as genomic sites that are under selection. It thus seems reasonable to expect that genome-based analyses with a comparable species coverage will be similarly insightful for coronaviruses. Also, additional diagnostic tools that target the entire species should be developed to complement existing tools optimized to detect individual pathogenic variants (a proactive approach). Technical solutions to this problem are already available; for example, in the context of multiplex PCR-based assays^42^. The costs for developing and applying (combined or separate) species- and virus-specific diagnostic tests in specific clinical and/or epidemiological settings may help to better appreciate the biological diversity and zoonotic potential of specific virus species and their members. Also, the further reduction of time required to identify the causative agents of novel virus infections will contribute to limiting the enormous social and economic consequences of large outbreaks. To advance such studies, innovative fundraising approaches may be required.

Although this Consensus Statement focuses on a single virus species, the issues raised apply to other species in the family and possibly beyond. A first step towards appreciation of this species and others would be for researchers, journals, databases and other relevant bodies to adopt proper referencing to the full taxonomy of coronaviruses under study, including explicit mentioning of the relevant virus species and the specific virus(es) within the species using the ICTV naming rules explained above. This naming convention is, regretfully, rarely observed in common practice, with mixing of virus and species names being frequently found in the literature (including by the authors of this Consensus Statement on several past occasions). The adoption of accurate virus-naming practices should be facilitated by the major revision of the virus species nomenclature that is currently being discussed by the ICTV and is being planned for implementation in the near future^43^. With this change in place, the CSG is resolved to address the existing significant overlap between virus and species names that complicates the appreciation and use of the species concept in its application to coronaviruses.
